# Investigating the Potential Protective Effect of Dog Ownership on Incident Disabling Dementia and Its Cost-Effectiveness

**DOI:** 10.3390/ijerph23070938

**Published:** 2026-07-22

**Authors:** Yu Taniguchi, Yoshiyuki Saito, Satoshi Seino, Toshiki Hata, Hiroki Mori, Erika Kobayasi

**Affiliations:** 1 Japan Environment and Children’s Study Programme Office, National Institute for Environmental Studies, 16-2 Onogawa, Tsukuba 305-8506, Japan; taniguchi.yu@nies.go.jp; 2 Department of Health Policy and Public Health, Graduate School of Pharmaceutical Sciences, Faculty of Pharmaceutical Sciences, The University of Tokyo, 7-3-1 Hongo, Bunkyo-ku, Tokyo 113-0033, Japan; 3 Institute of Well-Being, Yamagata University, 2-2-2 Iidanishi, Yamagata 990-9585, Japan; 4 Research Team for Social Participation and Healthy Aging, Tokyo Metropolitan Institute for Geriatrics and Gerontology (TMIG), 35-2 Sakae-cho, Itabashi-ku, Tokyo 173-0015, Japan; 5 Department of Nutrition and Life Science, School of Food and Nutritional Sciences, University of Shizuoka, 52-1 Yada, Suruga-ku, Shizuoka 422-8526, Japan

**Keywords:** dog, animals, dementia, potential causality, cost-effectiveness

## Abstract

**Highlights:**

**Public health relevance—How does this work relate to a public health issue?**

- Dementia prevention among older adults is a major public health challenge in aging societies.
- This study examined whether dog ownership is associated with the onset of disabling dementia in community-dwelling older adults.

**Public health significance—Why is this work of significance to public health?**

- Current dog ownership was associated with a lower risk of incident disabling dementia during follow-up.
- An exploratory cost-effectiveness evaluation suggested the potential public health value of promoting healthy aging through dog ownership-related behaviors.

**Public health implications—What are the key implications or messages for practitioners, policy makers and/or researchers in public health?**

- Dog ownership may represent a potentially modifiable lifestyle-related factor associated with healthy cognitive aging.
- Further studies using stronger causal inference approaches and more robust economic evaluations are needed to clarify the health effects of dog ownership.

**Abstract:**

This prospective study investigated the association of dog ownership with the onset of disabling dementia and mortality using propensity score weighting based on the physical, social, and psychological characteristics of dog owners, and then examined the potential causality of this relationship using an instrumental variable model. Additionally, we examined the costs and effects of dog ownership using cost-effectiveness analysis. Overall, 11,224 older adults selected using stratified and random sampling strategies in 2016 were analyzed. Dog ownership was defined as current, past, and never. Disabling dementia was defined according to a physician rating under the long-term care insurance system in Japan and mortality was ascertained by the Japanese national vital statistics during the approximately 7.5-year follow-up period. Current and past dog owners had an odds ratio (OR) of 0.53 (95% CI: 0.43–0.66) and 0.97 (0.86–1.09) of the onset of disabling dementia compared to never owners. ORs for mortality were 0.63 (0.51–0.79) in current dog owners and 0.88 (0.78–0.99) in past dog owners. Causal analysis showed that current dog owners (OR = 0.52, 95% CI: 0.27–0.98) and past dog owners (OR = 0.75, 95% CI: 0.55–1.03) had low ORs for incident dementia compared to never dog ownership. Corresponding values for mortality showed low ORs, but no significant effect on current dog owners (OR = 0.59, 95% CI: 0.35–1.01) and past dog owners (OR = 0.77, 95% CI: 0.60–1.00) compared to never owners. Results of cost-effectiveness analysis showed that dog ownership produced small but positive gains in quality-adjusted life years (QALYs) and was dominant (cost-saving and QALY-increasing) from the public payer perspective, reducing publicly financed medical and long-term care costs while increasing QALYs; when private dog-ownership costs were included in a scenario analysis, the incremental cost-effectiveness ratio (ICER) was ¥6,744,174 per QALY compared with no-dog status. This prospective study shows that dog ownership has a potential protective effect against the onset of disabling dementia in older adults. The study also revealed the potential of dog ownership to reduce public spending with regard to long-term care and medical expenditures.

## 1. Introduction

Accumulating evidence from studies of human–animal interaction indicates the presence of benefits in health outcomes among pet owners of various ages. In particular, numerous studies have demonstrated positive effects in humans in terms of daily physical activity [1,2,3], mental health [1,3,4], and protective effects against allergies [5,6]. Furthermore, animal-assisted therapy is widely known for its therapeutic benefit across a broad range of medical conditions [7,8,9,10,11] and a literature review provided Class IIa-IIb evidence (shown to be acceptable and useful) for recommending it to optimize healing environments [12,13].

With regard to the effects of human–animal interaction on older adults, we have reported that dog owners had a lower risk of incident frailty [14] and onset of disability [15] compared to never dog owners among community-dwelling elderly Japanese. A recent study [16] also showed that dog owners had a lower odds ratio for the onset of disabling dementia. As evidence supporting this association, dog owners tend to maintain cognitive function [17] and have higher levels of cognition and larger brain structure [18]. Moreover, a meta-analysis has reported a positive association between dog ownership and all-cause mortality and cardiovascular mortality [19]. Dog ownership may promote healthy aging by maintaining daily routines, providing companionship, and enhancing social engagement [20].

Epidemiological studies of people in Western countries [19] have shown an association between dog ownership and mortality, although one study with 3.5 years of follow-up [15] did not provide clear results due to the short observation period. Although previous studies [14,15,16,17,18,19] have examined the association between dog ownership and health outcomes among older adults after adjusting for confounding factors, this protective effect remains unclear.

According to the Integrative Model of Human–Animal Interactions [21], dog ownership may affect health through multiple interconnected biological, psychological, behavioral, and social pathways. This model provided the conceptual basis for our study by suggesting plausible mechanisms through which dog ownership could influence dementia risk. Based on this framework, we hypothesized that dog ownership may reduce the risk of disabling dementia in older adults. If a protective effect between dog ownership and reduced risk of dementia and mortality is in fact present, owning a dog may help prevent adverse health outcomes and thereby potentially reduce social security expenditures related to long-term care and medical treatment. The Human Animal Bond Research Institute reported that pet owners are estimated to visit the doctor less than non-pet owners on an annual basis [22], producing a cost savings of $15 billion [23]. We have reported that monthly long-term care costs for pet owners were approximately half those of non-pet owners [24]. To date, however, the cost-effectiveness of owning a dog and the potential economic benefits in medical treatment and long-term care have not been examined.

This study applied propensity score weighting and instrumental variable analysis to address the limitations of previous observational studies, such as residual confounding, reverse causality, and self-selection bias. This prospective 7.5-year study had three objectives: (1) to confirm the associations between dog ownership and the onset of dementia as well as mortality, using propensity score weighting based on the physical, social, and psychological characteristics of dog owners; (2) to investigate potential protective effect of dog ownership on incident disabling dementia and mortality, using an instrumental variable model; and (3) to evaluate the cost-effectiveness of dog ownership by examining the costs of owning a dog and its effects on long-term care and medical expenditures, using cost-effectiveness analysis. This study is a longitudinal study to combine causal inference methods with cost-effectiveness evaluation, and discusses countermeasures to address the ever-increasing social burden of dementia.

## 2. Materials and Methods

### 2.1. Study Population

The present study used data obtained from a community-based intervention trial initiated in 2016 [25]. In brief, 15,500 residents aged 65–84 years, representing approximately 10% of the older population of Ota City, Tokyo, Japan, were selected using stratified random sampling across all 18 districts as part of the Ota Genki Senior Project.

Potential participants were identified from the municipal resident registry and included community-dwelling adults aged 65 years or older who had not been certified as requiring long-term care. A self-administered baseline questionnaire was mailed to the 15,500 selected residents in June 2016. Of these, 11,925 individuals returned completed questionnaires, yielding a response rate of 76.9%. For the present analysis, participants were required to have available follow-up data on incident disabling dementia and all-cause mortality. After excluding individuals without these outcome assessments, the final analytical sample comprised 11,224 participants, corresponding to a valid response rate of 72.4%. The Ota Genki Senior Project was conducted in accordance with the ethical guidelines of the Tokyo Metropolitan Institute for Geriatrics and Gerontology and the principles of the Declaration of Helsinki. The questionnaire package included an explanation of the study objectives, emphasized the voluntary nature of participation, and assured participants that all responses would be analyzed anonymously. Informed consent was obtained from all participants, with the return of the completed questionnaire regarded as consent to participate. Participants were prospectively followed for 7.5 years through linkage with the municipal long-term care insurance database and municipal resident registries, enabling ascertainment of incident disabling dementia and all-cause mortality. The study protocol was approved by the Ethics Committee of the Tokyo Metropolitan Institute for Geriatrics and Gerontology on 1 June 2016 (Approval No. 8) and 18 June 2018 (Approval No. 22).

### 2.2. Definition of Dog Ownership

Participants were asked if they lived with a pet (current, past, or never). Those with current or past pet ownership experience were asked to indicate the pet species (dog, cat, or other) [14,15,26]. Responses were used to classify dog ownership as “current”, “past” or “never” in the baseline survey in 2016.

### 2.3. Measurement of Dementia and All-Cause Mortality

Incident disabling dementia was identified using the nationally standardized dementia assessment conducted within Japan’s long-term care insurance (LTCI) system, which covers the majority of older adults with dementia. Under this system, physicians are required by the Ministry of Health, Labour and Welfare to evaluate cognitive impairment using an observer-based assessment [27]. The evaluation is completed using a standardized form that assesses chronic medical conditions and activities of daily living and is uniformly implemented across Japan [28,29].

The dementia scale classifies individuals into five categories: no dementia; level I (dementia with preserved independence in daily life); level II (dementia requiring minimal supervision despite relative independence); level III (dementia requiring partial assistance because of communication and functional difficulties); and level IV (severe dementia requiring comprehensive care owing to marked communication impairment). Consistent with previous studies [28,30,31,32,33], disabling dementia was defined as a dementia level of II or higher. This threshold was selected because individuals classified at level II or above are eligible for LTCI benefits, including institutional care, home care, respite services, day-care services, and assistive equipment [34,35,36]. We also examined local registries to ascertain deaths from any cause and linked these data with Japanese national vital statistics [37,38]. We checked for incident disabling dementia or death until December 2023, giving a follow-up period of approximately 7.5 years, and extracted the date on which participants were first classified as having disabling dementia or death.

### 2.4. Demographic and Health Characteristics

Covariates included in our estimation equations included demographic and health characteristics, namely sex, age, household size, marital status, educational attainment, equivalent income, employment, history of chronic diseases, history of hospitalization and falls during the past year, alcohol drinking, smoking status, sleep duration, food variety, higher level functional capacity, mobility limitation, BMI, motor fitness scale, exercise habit, frailty status, interaction with neighbors, social isolation, trust in neighbors, frequency of going outdoors, subjective happiness, self-rated health, Geriatric Depression Scale [GDS]-5 score, and WHO-5 score.

Health-related characteristics included several clinically relevant chronic conditions: hypertension, hyperlipidemia, heart disease, stroke, diabetes mellitus, chronic respiratory disease, and cancer. Participants reported whether they had ever been diagnosed with each condition by a physician (yes/no). Dietary diversity was evaluated using a dietary variety score based on the weekly consumption of 10 food groups (meat, fish and shellfish, eggs, milk, soybean products, green and yellow vegetables, potatoes, fruit, seaweed, and fats/oils). Scores ranged from 0 to 10, with higher values indicating greater dietary variety [39]. Higher-level functional capacity was measured using the Tokyo Metropolitan Institute of Gerontology Index of Competence (TMIG-IC), which assesses three domains of Lawton’s competence model: instrumental self-maintenance, intellectual activity, and social role [38]. Total scores range from 0 to 13, with lower scores reflecting poorer functional capacity. Mobility limitation was defined as self-reported difficulty in walking 0.4 km (one-quarter of a mile) or climbing 10 stairs without resting [40]. Motor function was assessed using the Motor Fitness Scale, a 14-item instrument measuring basic motor abilities. Scores range from 0 to 14, with higher scores indicating better motor performance [41]. Frailty was evaluated using a modified version of the Kaigo-Yobo Checklist. Total scores range from 0 to 15, and participants scoring >4 were classified as frail [42]. Participants also reported the types of exercise they performed at least once per week, including walking, running, muscle-strengthening exercise, stretching, swimming, cycling, yoga, and other activities. Based on these responses, participants were categorized as having either an exercise habit or no exercise habit. Neighborhood social interaction was dichotomized into high (close relationships or conversations with neighbors) and low (greetings only or no interaction). Social isolation was determined from the frequency of both face-to-face and non-face-to-face contact with non-cohabiting children, relatives, friends, and neighbors. Participants reporting contact less than once per week were classified as socially isolated [43]. Subjective happiness was assessed using four response categories: happy, rather happy, rather unhappy, and unhappy. Depressive symptoms were measured using the 5-item Geriatric Depression Scale (GDS-5), with higher scores indicating greater depressive symptomatology [44]. Psychological well-being was evaluated using the World Health Organization-Five Well-Being Index (WHO-5), which yields scores ranging from 0 to 100, with lower scores representing poorer positive mood and vitality [45].

### 2.5. Statistical Analysis

Baseline demographic and health characteristics were summarized according to dog ownership status. To evaluate the association between dog ownership and the risks of incident disabling dementia and all-cause mortality while accounting for differences in baseline characteristics, we applied inverse probability of treatment weighting based on propensity scores within a logistic regression framework. This approach balanced measured baseline covariates between current owners and past or never owners by assigning weights to each individual. Robust variance estimators were used to calculate standard errors for the estimated associations between dog ownership and each outcome. Propensity score weights were derived using variables previously identified as predictors of dog ownership among community-dwelling older Japanese adults [26]. These variables included sex, age, household size, equivalent household income, employment status, histories of chronic respiratory disease and cancer, hospitalization during the previous year, smoking status, frailty, interaction with neighbors, trust in neighbors, and WHO-5 score. The follow-up period was adjusted. We then examined the potential protective effect of dog ownership on incident disabling dementia and mortality using an instrumental variable [46] with population density of participants’ residential area as an instrument, based on the consideration that this variable was correlated with dog ownership only. Population density is associated with dog ownership patterns because urban environments generally have lower dog ownership rates due to housing conditions and lifestyle factors [47]. In contrast, evidence regarding the relationship between population density and cognitive health has been inconsistent, with previous studies reporting negative, null, and positive associations after accounting for individual-level characteristics. We have cited these studies to better support the plausibility of the exclusion restriction assumption. This variability suggests that population density itself may not directly influence the risk of dementia. Covariates in the instrumental variable were overlapped in an inverse probability of treatment-weighted logistic regression model.

To synthesize clinical effects into costs and quality-adjusted life years (QALYs), we used a cohort state-transition (Markov) model with three mutually exclusive health states, namely healthy, dementia, and death. Cycle length was 1 month and the time horizon was 48 months. We adopted a Japanese public payer perspective, in which costs consist of direct medical and long-term care expenditures financed by public health insurance and the long-term care insurance (LTCI) system, and discounted both costs and QALYs at 2% per annum. Pet-ownership expenses are private, out-of-pocket spending by households. In the base-case cost-effectiveness analysis, we reported total costs from a public payer perspective, including only publicly financed medical and long-term care expenditures and excluding private dog-ownership expenses. In a scenario analysis, we additionally included private, out-of-pocket dog-ownership expenses to reflect the combined financial burden borne by the health and long-term care system and households. The initial cohort comprised 1000 healthy individuals. In intuitive terms, the model follows this hypothetical cohort of initially healthy older adults and updates on a monthly basis how many remain healthy, develop disabling dementia, or die under each scenario (no dog vs. dog ownership). For each health state, we attached monthly costs (medical and long-term care) and health-state utilities to calculate total discounted costs and QALYs over 48 months, with a half-cycle correction. Because the model used a monthly cycle, the annual health-state utilities were converted to the QALYs accrued within each cycle by multiplying by one-twelfth (1/12), consistent with the monthly accrual of costs. We derived monthly transition probabilities for the no-dog group from the 7.5-year cumulative risks observed in this cohort, and used these to define transitions from healthy to dementia, healthy to death, and from dementia to death. Post-dementia mortality (the dementia-to-death transition) was assumed to be identical across all scenarios, so that dog ownership was modeled to reduce dementia incidence but not post-dementia survival. Specifically, we defined a dog (GEE) scenario based on odds ratios from inverse probability of treatment-weighted generalized estimating equation models. Because the Markov model operates on probabilities rather than odds, each odds ratio was converted to a risk ratio using the baseline cumulative risk over the model horizon; the resulting risk ratio was applied at the cumulative level and then re-expressed as a monthly transition probability. Per-cycle (monthly) costs were set to ¥30,000 for healthy and ¥157,000 for dementia, with reference to Japanese estimates of medical and long-term care expenditures for dementia and non-dementia older adults [48,49,50]. The assumed dog ownership cost (¥25,000 per month) was consistent with reported pet-related expenditures and previous analyses of pet ownership and medical/long-term care costs among older Japanese [24]. Health-state utilities were assumed to be 0.85 for healthy and 0.50 for dementia, with reference to published EQ-5D-based utilities in older adults and people with dementia, and 0.00 for death [51,52]. Outcomes were total discounted costs and QALYs (expected values), incremental cost, incremental QALY, and the incremental cost-effectiveness ratio (ICER). The ICER was defined as the difference in total costs divided by the difference in total QALYs between the dog-ownership (Dog (GEE)) and no-dog scenarios. For the budget impact (BI) analysis, we scaled the 1000-person cost difference to the Japanese older population (36 million). We did not assume universal dog ownership; instead, we applied an uptake rate of 13.4%, which was calculated by multiplying the observed current dog-ownership prevalence in this cohort (8.6%) by 1.56 based on a survey of dog and cat ownership in Japan that also captured the proportion of older adults who expressed a desire to own a dog [53]. In the BI analysis, dog-ownership costs were treated as an intervention-like cost borne by households, and added to public medical and long-term care costs to capture the overall increase in spending associated with greater dog ownership. In addition, we calculated gross dementia-related cost offsets as the difference in total medical and long-term care costs between the dog-ownership scenario (Dog (GEE)) and the no-dog scenario, excluding dog-ownership costs, to describe the potential savings in health and long-term care expenditures. An uncertainty analysis of all estimations was implemented as a first-order Monte Carlo (stochastic) simulation of the Markov model with binomial sampling of transitions for each cycle (10,000 simulations per scenario); because the model parameters were held fixed, this represents first-order rather than parameter uncertainty. From these simulations, we obtained empirical 95% confidence intervals (CIs) for incident dementia counts, incremental costs, and incremental QALYs. We did not report a confidence interval for the ICER because ICER interval estimates are unstable when the incremental effect approaches zero; instead, decision uncertainty was summarized using a cost-effectiveness acceptability curve and one-way sensitivity analyses (Supplementary Figures S1 and S2). Statistical analyses were conducted using Stata SE (version 18; Stata Corp, College Station, TX, USA), SPSS (version 23.0; IBM Corp, Armonk, NY, USA), and Python (version 3.7.4).

## 3. Results

### 3.1. Sample Characteristics

The 11,224 participants in the baseline survey consisted of 963 (8.6%) current dog owners, 2539 (22.6%) past dog owners, and 7722 (68.8%) never dog owners (Table 1). The mean (standard deviation; SD) age of participants was 74.2 (5.4) years, and 51.5% were women. Mean household size was 2.3 (1.1); 67.7% were married; 73.4% had an educational attainment of high school, college, university, or graduate school; 33.0% had a yen-equivalent income of 1,000,000–2,500,000 yen and 25.7% had 2,500,000–4,000,000 yen; and 26.9% had a job.

### 3.2. Associations Between Dog Ownership and the Onset of Dementia and Mortality

During the follow-up period of approximately 7.5-years, 1989 (17.7%) had disabling dementia and 1808 (16.1%) died. Among current, past, and never dog owners, 12.8%, 18.5%, and 18.1% had disabling dementia, and 12.3%, 15.7%, and 16.7% had incident death, respectively. Results of the inverse probability of treatment-weighted logistic regression model showed that current dog owners had an OR of 0.53 (95% CI: 0.43–0.66) and past dog owners had an OR of 0.97 (95% CI: 0.86–1.09) of disabling dementia compared to never owners (Table 2). For all-cause mortality, current dog owners had an OR of 0.63 (95% CI: 0.51–0.79) and past dog owners had an OR of 0.88 (95% CI: 0.78–0.99) compared to never owners.

### 3.3. Potential Protective Effect of Dog Ownership on Incident Dementia and Mortality

Results in an instrumental variable model using population density as the exogenous instrument showed that current dog owners had a significantly low OR (OR = 0.52, 95% CI: 0.27–0.98) and past dog owners had a low OR (OR = 0.75, 95% CI: 0.55–1.03) for incident dementia compared to never dog ownership (Table 3). The minimum eigenvalue statistic was 31.9, indicating that the instrument was reasonable. Corresponding values for mortality showed low ORs, but no statistically significant effect was found for current (OR = 0.59, 95% CI: 0.35–1.01) or past dog owners (OR = 0.77, 95% CI: 0.60–1.00) compared to never owners.

### 3.4. Cost-Effectiveness of Dog Ownership for Medical and Long-Term Care Expenditures

Over the 48-month time horizon, from the public payer perspective, the dog-ownership scenario (Dog (GEE)) yielded lower medical and long-term care costs and higher QALYs than the no-dog scenario (Table 4). In the base-case analysis, which considered only publicly financed medical and long-term care expenditures and excluded private dog-ownership costs, dog ownership was dominant: it reduced these public costs by ¥122,717,829 (95% CI ¥30,312,493 to ¥211,632,695) and increased QALYs by 142.4 (95% CI 78.9 to 207.2) per modeled cohort of 1000 individuals compared with no dog. When private, out-of-pocket dog-ownership expenses were additionally included in a scenario analysis, the incremental cost was ¥960,372,661 (95% CI ¥867,603,762 to ¥1,056,062,973), giving an incremental cost-effectiveness ratio (ICER) of ¥6,744,174 per QALY; this exceeds the commonly cited Japanese threshold of approximately ¥5,000,000 per QALY suggested by formal Health Technology Assessment (HTA) implementation and willingness-to-pay studies [54,55], and the probability of being cost-effective at that threshold was approximately 7% (Supplementary Figures S1 and S2). In the scenario including private dog-ownership costs, the mean 4-year budget impact was approximately ¥4.63 trillion (95% CI ¥4.19 to ¥5.09 trillion), assuming a dog-ownership uptake rate of 13.4%. From the public payer perspective, dog ownership could reduce dementia-related medical and long-term care expenditures by approximately ¥592 billion over four years at a dog-ownership prevalence of 13.4%.

## 4. Discussion

A previous study using propensity scoring and an approximately 4-year follow-up period demonstrated that dog ownership has a protective effect on incident disabling dementia compared to non-ownership [16]. The present study suggests that dog ownership has a protective effect on incident disabling dementia and mortality during an approximately 7.5-year follow-up period. Dog owners had ORs of 0.53 (95% CI: 0.43–0.66) and 0.63 (95% CI: 0.51–0.79) for incident disabling dementia and mortality using propensity score weighting based on physical, social, and psychological characteristics compared to never owners. Moreover, this study identified a potential protective effect of dog ownership on incident disabling dementia and mortality using an instrumental variable model, with ORs of dog owners of 0.52 (95% CI: 0.27–0.98) and 0.59 (95% CI: 0.35–1.01) compared to never owners. The present study also revealed the potential of dog ownership to reduce long-term care and medical expenditures from a public payer perspective. Cost-effectiveness analysis showed that dog ownership was dominant (cost-saving and QALY-increasing) from the public payer perspective, reducing publicly financed medical and long-term care costs while increasing QALYs; when private dog-ownership costs were included in a scenario analysis, the incremental cost-effectiveness ratio (ICER) was ¥6,744,174 per QALY compared with no dog.

A number of mechanisms are reported to underlie the protective effect of dog ownership on incident disabling dementia. Friedman et al. reported that deterioration in cognitive function with age was slower in dog owners than non-owners after controlling for age and comorbidities [17]. McDonough et al. reported that pet ownership was related to higher levels of cognition and larger brain structures, and that these effects were largest in dog owners [18]. Our present finding that dog ownership is causally related to a lower risk of dementia supports the results of these previous studies. The most recent research has also shown that owning a dog can increase walking time and social contact [56]. Dog walking is categorized as a physical activity with three metabolic equivalents of moderate intensity [57], and dog owners who walk their dog are 2.5 times more likely to achieve at least moderate-intensity physical activity [58]. Dog walking has also been implicated as a means of increasing opportunities for social interaction and improving psychological health for older adults [59]. Physical inactivity, social isolation, and mental health are known risk factors for the development of dementia [60]. From this evidence, we believe that maintaining and improving these factors through dog walking might reduce the risk of dementia.

As another possible mechanism of these risk factors in the development of dementia, Handlin et at. reported that short-term interaction between a dog and its owner increased the dog’s oxytocin levels [61]. Nagasawa et al. reported that gazing behavior from dogs increased urinary oxytocin concentrations in owners, which consequently facilitated owners’ affiliation and increased oxytocin concentration in dogs [62]. Oxytocin is known for being the hormone of love and plays a key role in social behaviors. Oxytocin has also been shown to play a significant role in lipid metabolism, and it is possible that exposure to dogs may have a positive effect on lipid metabolism for dog owners, which plays a major role in the pathogenesis of Alzheimer’s disease. Furthermore, exposure to dogs also affects gut microbiota for dog owners. Living with dogs leads to more contact with soil, plants, and other dogs during dog walks, which may increase the likelihood of various bacteria being brought into the home and potentially increase the diversity of the gut microbiota. Gupta mentioned that infants living with pets had a higher diversity of microbes and potentially beneficial bacterial groups in the gut than infants without pets [63]. Dog ownership may have positive effects on the autonomic nervous system and/or immune system through beneficial microbes, thereby reducing the risk of dementia. Based on the “Old Friends” hypothesis, it is possible that living without dogs may increase the risk of dementia because people are deprived of the benefits mediated by microbes.

In this study, while dog ownership showed a significant association with mortality, the protective effect was not statistically significant. A meta-analysis identified a positive association between dog ownership and cardiovascular mortality [19]. This result was marginal and did not preclude a protective effect of dog ownership on mortality and cardiovascular mortality. Further, the observation period of 7 years in our study may have been short. Future studies should extend the observation period and reconfirm the protective effect of dog ownership on all-cause mortality and cardiovascular mortality.

When synthesized in a 48-month state-transition model from a Japanese payer perspective, dog ownership generated health gains while reducing publicly financed medical and long-term care costs, and was dominant in the base-case analysis. When private, out-of-pocket dog-ownership expenses were included in a scenario analysis, the ICER (¥6,744,174 per QALY) exceeded conventional Japanese thresholds, so the assessed value of dog ownership depends on whether private ownership costs are attributed to the health system. Regarding health economics analysis, the budget impact depends on uptake and financing. When high dog ownership prevalence and out-of-pocket pet expenses are modeled as an “intervention cost,” health system budgets may face additional spending even when ICERs are attractive. Policy-relevant levers include targeting (e.g., physically inactive or socially isolated subgroups), partial subsidies for community dog walking programs, or integrating animal-assisted social participation initiatives that capture health benefits without assuming universal private ownership costs. Given that the societal cost of dementia in Japan has been estimated at JPY 14.5 trillion annually, with informal care and long-term care accounting for a large share [48,49,50], even modest preventive effects may translate into substantial fiscal and societal gains. Treating private costs such as dog care fees as a separate category would have the potential to save ¥592 billion in medical and nursing care costs socially. Therefore, rather than using the budget for dog care itself, it may be more realistic for the government to allocate it to supporting current companion animal owners and increasing the number of people who want to keep a companion animal. Our health economic analysis was primarily conducted from a public payer perspective and captured changes in publicly financed medical and long-term care expenditures. Dog-ownership costs represent private consumption and may have broader macroeconomic benefits through pet-related industries; these wider societal effects were beyond the scope of the present payer-focused evaluation. As an upper-bound illustrative scenario, if the observed association between dog ownership and reduced dementia incidence were hypothetically applied to all newly incident cases nationwide, the corresponding reduction in dementia-related medical and long-term care expenditures could approach ¥5–6 trillion over four years. While such a scenario is not intended to represent a realistic policy projection, it underscores the potentially large macroeconomic impact of preventive interventions targeting modifiable social determinants of health.

This study has several main strengths. First, our sample of over 11,000 community-dwelling older Japanese is large and the data were derived from a survey with a response rate of 76.9%. Second, our analysis included a wide range of demographics and health characteristics. This enabled us to examine the associations and protective effects of dog ownership on disabling dementia and mortality, as well as the net of baseline demographics and health. Third, data on incident disabling dementia were derived from the LTCI system database, and data on cause of death was linked with the Japanese national vital statistics. Thus, the universal LTCI system and national vital statistics in Japan enabled us to link dog ownership with incident disabling dementia and mortality.

This study also has some limitations. First, the proportion of dog and cat owners is smaller in Japan than in Western countries. The lifestyle of owners with their dogs and the breeds of dogs might differ in Japan compared with other countries. Accordingly, it is necessary to confirm whether the potential protective association between dog ownership and incident dementia, as well as its cost-effectiveness, can also be observed in other countries. Second, dog ownership was assessed only at baseline, and changes in ownership status during the 7.5-year follow-up could not be captured. Consequently, some current owners may have stopped owning dogs, whereas some past or never owners may have subsequently acquired a dog. We were also unable to collect information on the frequency of dog care. Together with changes in dog ownership status during follow-up, this may have further attenuated the observed association between current dog ownership and incident dementia. Furthermore, individuals with better cognitive and physical functioning may have been more likely to own and continue caring for a dog than those experiencing early cognitive or physical decline. Although we attempted to reduce this bias using propensity score weighting and instrumental variable analysis, reverse causation cannot be completely excluded. In addition, the COVID-19 pandemic occurred during the follow-up period of this study, which may have increased disability and death due to coronavirus [64]. Considering the possibility that dog owners maintained their lifestyles even during the COVID-19 pandemic, the pandemic may have exacerbated the effect on health outcomes observed between dog owners and past and never owners. Therefore, the observed associations should be interpreted as evidence consistent with a potential protective effect of dog ownership rather than definitive proof of causality. Third, although propensity score weighting substantially improved covariate balance, residual confounding due to unmeasured or imperfectly measured factors cannot be completely excluded. In addition, some variables included in the propensity score model, such as frailty status, social interaction, and psychological well-being, may partially lie on the causal pathway between dog ownership and incident dementia. Adjustment for these variables may have attenuated the estimated association through overadjustment. Therefore, the results of the propensity score-weighted analyses should be interpreted with appropriate caution. Fourth, we used the population density of the participants’ residential area as the instrumental variable. Although descriptive statistics consistently show lower dog ownership rates in high-density urban areas than in rural settings, the exclusion restriction assumption underlying instrumental variable analysis cannot be empirically verified in an observational study. Population density may also be associated with other environmental or socioeconomic characteristics that could influence dementia risk. Therefore, our instrumental variable analysis should be interpreted as providing evidence consistent with a potential protective effect rather than definitive proof of causality. Future research should explore and validate more robust instrumental variables to better establish the protective effect of dog ownership and health outcomes. Fifth, we suggest that while the government should allocate funds to support current companion animal owners, measures to address the social issues arising from animal farming are also necessary at the same time. Sixth, our cost-effectiveness analysis included several limitations, i.e., assumptions when mapping ORs to monthly transition risks and simplified cost/utility inputs that may underestimate or overestimate offsets. Future studies should incorporate caregiver costs, heterogeneity by living arrangement, and longer time horizons.

Finally, our findings add to the growing body of evidence supporting the potential health benefits of dog ownership. Although previous studies have reported associations between dog ownership and various physical, psychological, and social health outcomes, evidence regarding incident disabling dementia has remained limited. By applying both propensity score weighting and an instrumental variable approach, our study extends previous observational research and provides evidence consistent with a potential protective effect of current dog ownership on incident disabling dementia. However, because the validity of the instrumental variable relies on assumptions that cannot be fully verified, these findings should not be interpreted as definitive proof of causality. Further longitudinal studies using stronger causal inference designs and more robust instrumental variables are needed to confirm these findings and better understand the underlying mechanisms.

## 5. Conclusions

In summary, this prospective study suggests that dog ownership has a protective effect on incident disabling dementia in older adults. The study also revealed the potential of dog ownership to reduce long-term care and medical expenditures from a public payer perspective, where it was dominant (cost-saving with greater QALYs), although its cost-effectiveness when private ownership costs are included depends on the willingness-to-pay threshold. Living with a dog may be recommended as a component of health promotion policy and may have an important role to play in policies aimed at achieving a stable economy.

## Figures and Tables

**Table 1:** Baseline demographic and health characteristics in older community-dwelling Japanese by dog ownership experience.

Variable	Experience with Dog Ownership			
	Current (*n* = 963, 8.6%)	Past (*n* = 2539, 22.6%)	Never (*n* = 7722, 68.8%)	*p*-Value
Sex (%, female)	54.4	52.4	50.9	0.070
Age (%)				
65–74 years	59.0	46.8	46.7	<0.001
75–84 years	41.0	53.2	53.3	
Household size	2.7 (1.2)	2.4 (1.2)	2.2 (1.1)	<0.001
Marital status (%)				
Married	74.0	69.8	65.3	<0.001
Divorced	5.8	5.6	6.3	
Widowed	18.1	19.7	19.7	
Single	2.1	4.9	8.6	
Educational attainment (%)				
Elementary school	0.8	1.1	1.7	<0.001
Middle school	17.2	17.9	25.7	
High school	37.8	36.9	39.1	
College, university, or graduate school	42.0	42.4	31.5	
Other	2.2	1.7	1.9	
Equivalent income (%)				
<1,000,000 yen	6.5	6.5	7.6	<0.001
1,000,000–2,500,000 yen	26.2	29.1	35.1	
2,500,000–4,000,000 yen	22.8	25.1	26.3	
≥4,000,000 yen	26.4	23.9	17.3	
Unknown	18.0	15.5	13.6	
Employment (%, present)	35.4	29.4	26.5	<0.001
Chronic disease (%)				
Hypertension	55.3	53.9	54.4	0.751
Hyperlipidemia	44.5	42.5	41.9	0.312
Heart disease	21.5	22.7	21.8	0.623
Stroke	8.4	7.7	7.6	0.734
Diabetes mellitus	18.5	18.3	18.9	0.797
Bone and joint disease	30.1	32.0	31.9	0.521
Respiratory lung disease	13.8	17.1	14.6	0.007
Cancer	18.0	17.8	16.2	0.111
Hospitalization during the past year (%)	14.8	13.3	12.2	0.040
Fall during the past year (%)	15.3	16.3	14.5	0.073
Alcohol consumption status (%)				
Current	57.4	56.4	54.3	0.014
Past	5.8	8.6	8.4	
Never	36.7	35.1	37.4	
Smoking status (%)				
Current	13.9	11.7	12.7	0.016
Past	33.9	35.2	31.9	
Never	52.2	53.1	55.4	
Sleep duration (min)	397 (69)	396 (73.0)	396 (74)	0.971
Food variety (score)	3.0 (2.2)	3.3 (2.2)	3.2 (2.2)	0.010
TMIG-IC (score)	11.6 (1.8)	11.6 (1.9)	11.3 (1.9)	<0.001
Mobility limitation (%)	25.5	27.7	30.5	<0.001
BMI (kg/m^2^)	23.0 (3.3)	22.6 (3.2)	22.7 (3.2)	<0.001
Motor fitness scale (score)	11.0 (3.1)	10.9 (3.2)	10.5 (3.3)	<0.001
Regular exercise habit (%)	77.7	76.1	72.7	<0.001
Frailty (%)	20.2	21.2	24.7	<0.001
Interaction with neighbors (%)				
Significant relationship	25.5	24.6	22.5	<0.001
Conversation	40.6	38.3	36.8	
Exchange of greetings only	29.7	31.6	33.3	
No social contact	4.2	5.5	7.4	
Social isolation (%)	24.0	28.1	34.2	<0.001
Trust in neighbors (%, yes)	81.1	79.0	76.0	<0.001
Frequency of going outdoors (%)				
At least once a day	81.0	73.1	74.3	<0.001
Once every 2–3 days	14.7	19.5	18.3	
Less than once a week	4.3	7.4	7.4	
Subjective happiness (%)				
Happy, rather happy	95.0	94.9	93.1	<0.001
Rather unhappy, unhappy	5.0	5.1	6.9	
Self-rated health (%)				
Excellent, good	83.0	83.0	80.2	<0.001
Fair, poor	17.0	17.0	19.8	
GDS Short-version (score)	1.2 (1.2)	1.3 (1.3)	1.3 (1.3)	0.045
WHO-5 (score)	63.4 (22.9)	62.7 (23.2)	60.2 (24.3)	<0.001

BMI, body mass index; TMIG-IC, Tokyo Metropolitan Institute of Gerontology Index of Competence; GDS, Geriatric Depression Scale; WHO-5, World Health Organization Five Well-Being Index.

**Table 2:** Independent associations between experience with dog ownership and incident dementia and mortality among community-dwelling older Japanese.

Independent Variable	Incident Dementia	OR in GEE (95% CI)	Incident Death	OR in GEE (95% CI)
Experience with dog ownership				
Never (*n* = 7722, 68.8%) §	1396/7722 (18.1%)	1	1292/7722 (16.7%)	1
Past (*n* = 2539, 22.6%)	470/2539 (18.5%)	0.97 (0.86–1.09)	398/2539 (15.7%)	0.88 (0.78–0.99)
Current (*n* = 963, 8.6%)	123/963 (12.8%)	0.53 (0.43–0.66)	118/963 (12.3%)	0.63 (0.51–0.79)

§ reference group. GEE, generalized estimating equation; OR, odds ratio; CI, confidence interval. Analysis was weighted by the inverse of propensity score in the GEE. Weight was calculated based on sex, age, household size, equivalent income, employment, history of lung respiratory disease and cancer, history of hospitalization during the past year, smoking status, frailty status, interaction with neighbors, trust in neighbors, and WHO-5. Adjusted for follow-up period.

**Table 3:** Potential causal relationship of dog ownership with incident dementia and mortality.

	Incident Dementia	Incident Death
	OR in Instrumental Variables (95% CI)	OR in Instrumental Variables (95% CI)
Experience with dog ownership		
Past	0.75 (0.55–1.03)	0.77 (0.60–1.00)
Current	0.52 (0.27–0.98)	0.59 (0.35–1.01)

Reference group was never dog ownership. Instrumental variables with population density of the residential area as third variable were run in adjusted model: follow-up year, sex, age, household size, equivalent income, employment, history of lung respiratory disease and cancer, history of hospitalization during the past year, smoking status, frailty status, interaction with neighbors, trust in neighbors, and WHO-5.

**Table 4:** Cost-effectiveness and budget impact of dog-ownership scenarios over 48 months.

Scenario	Cost	QALY	Dementia Cases	ICER	Budget Impact	ΔCost per 1000 Savings	National Savings
No dog §	1,653,136,365	2,873.37	162				
Dog (GEE)	2,613,509,026	3,015.77	96	6,744,174	4,632,837,717,372	122,717,829	591,990,804,911

§ Reference was no dog. Budget impact was assuming an ownership rate of 13.4%. The cost offset per 1000 persons (“ΔCost per 1000 savings”) was calculated assuming zero pet-ownership cost and was estimated over 4 years.

## Data Availability

The data of a community-wide intervention trial (Ota Genki Senior Project) in Ota City contains sensitive participant information and cannot be released publicly due to ethicolegal restrictions imposed by the Ethics Committee of Tokyo Metropolitan Institute of Gerontology. The datasets generated during and/or analyzed during the current study are available from the corresponding author on reasonable request. Long-term storage data is available upon reasonable request to Yu Taniguchi (taniguchi.yu@nies.go.jp) and Hiroki Mori (h_mori@tmig.or.jp).

## Supplementary Materials

The following supporting information can be downloaded at: https://www.mdpi.com/article/10.3390/ijerph23070938/s1, Figure S1: Cost-effectiveness acceptability curve for the base-case comparison of dog ownership; Figure S2: One-way sensitivity analysis of the incremental cost-effectiveness ratio.

## References

[1] Martins C.F., Soares J.P., Cortinhas A., Silva L., Cardoso L., Pires M.A., Mota M.P. (2023). Pet’s influence on humans’ daily physical activity and mental health: A meta-analysis. Front. Public Health.

[2] Christian H.E., Westgarth C., Bauman A., Richards E.A., Rhodes R.E., Evenson K.R., Mayer J.A., Thorpe R.J. (2013). Dog ownership and physical activity: A review of the evidence. J. Phys. Act. Health.

[3] Gee N.R., Mueller M.K. (2019). A Systematic Review of Research on Pet Ownership and Animal Interactions among Older Adults. Anthrozoös.

[4] Rodriguez K.E., Greer J., Yatcilla J.K., Beck A.M., O’Haire M.E. (2020). The effects of assistance dogs on psychosocial health and wellbeing: A systematic literature review. PLoS ONE.

[5] Pinot de Moira A., Strandberg-Larsen K., Bishop T., Pedersen M., Avraam D., Cadman T., Calas L., Casas M., de Lauzon Guillain B., Elhakeem A. (2022). Associations of early-life pet ownership with asthma and allergic sensitization: A meta-analysis of more than 77,000 children from the EU Child Cohort Network. J. Allergy Clin. Immunol..

[6] Takkouche B., Gonzalez-Barcala F.J., Etminan M., Fitzgerald M. (2008). Exposure to furry pets and the risk of asthma and allergic rhinitis: A meta-analysis. Allergy.

[7] Maber-Aleksandrowicz S., Avent C., Hassiotis A. (2016). A Systematic Review of Animal-Assisted Therapy on Psychosocial Outcomes in People with Intellectual Disability. Res. Dev. Disabil..

[8] O’Haire M.E. (2013). Animal-assisted intervention for autism spectrum disorder: A systematic literature review. J. Autism Dev. Disord..

[9] Kamioka H., Okada S., Tsutani K., Park H., Okuizumi H., Handa S., Oshio T., Park S.J., Kitayuguchi J., Abe T. (2014). Effectiveness of animal-assisted therapy: A systematic review of randomized controlled trials. Complement. Ther. Med..

[10] Lundqvist M., Carlsson P., Sjödahl R., Theodorsson E., Levin L. (2017). Patient benefit of dog-assisted interventions in health care: A systematic review. BMC Complement. Altern. Med..

[11] Villafaina-Domínguez B., Collado-Mateo D., Merellano-Navarro E., Villafaina S. (2020). Effects of Dog-Based Animal-Assisted Interventions in Prison Population: A Systematic Review. Animals.

[12] Marcus D.A. (2013). The science behind animal-assisted therapy. Curr. Pain Headache Rep..

[13] Halm M.A. (2008). The healing power of the human-animal connection. Am. J. Crit. Care.

[14] Taniguchi Y., Seino S., Nishi M., Tomine Y., Tanaka I., Yokoyama Y., Ikeuchi T., Kitamura A., Shinkai S. (2019). Association of Dog and Cat Ownership with Incident Frailty among Community-Dwelling Elderly Japanese. Sci. Rep..

[15] Taniguchi Y., Seino S., Headey B., Hata T., Ikeuchi T., Abe T., Shinkai S., Kitamura A. (2022). Evidence that dog ownership protects against the onset of disability in an older community-dwelling Japanese population. PLoS ONE.

[16] Taniguchi Y., Seino S., Ikeuchi T., Hata T., Shinkai S., Kitamura A., Fujiwara Y. (2023). Protective effects of dog ownership against the onset of disabling dementia in older community-dwelling Japanese: A longitudinal study. Prev. Med. Rep..

[17] Friedmann E., Gee N.R., Simonsick E.M., Kitner-Triolo M.H., Resnick B., Adesanya I., Koodaly L., Gurlu M. (2023). Pet ownership and maintenance of cognitive function in community-residing older adults: Evidence from the Baltimore Longitudinal Study of Aging (BLSA). Sci. Rep..

[18] McDonough I.M., Erwin H.B., Sin N.L., Allen R.S. (2022). Pet ownership is associated with greater cognitive and brain health in a cross-sectional sample across the adult lifespan. Front. Aging Neurosci..

[19] Kramer C.K., Mehmood S., Suen R.S. (2019). Dog Ownership and Survival: A Systematic Review and Meta-Analysis. Circ. Cardiovasc. Qual. Outcomes.

[20] Monks S., Clark A. (2024). The role of pets in the lives of people with dementia: A scoping review. Aging Ment. Health.

[21] Leconstant C., Spitz E. (2022). Integrative Model of Human-Animal Interactions: A One Health–One Welfare Systemic Approach to Studying HAI. Front. Vet. Sci..

[22] Human Animal Bond Research Institute (2023). Health Care Cost Savings of Pet Ownership.

[23] Headey B., Grabka M.M. (2007). Pets and Human Health in Germany and Australia: National Longitudinal Results. Soc. Indic. Res..

[24] Taniguchi Y., Yokoyama Y., Ikeuchi T., Mitsutake S., Murayama H., Abe T., Seino S., Amano H., Nishi M., Hagiwara Y. (2023). Pet ownership-related differences in medical and long-term care costs among community-dwelling older Japanese. PLoS ONE.

[25] Seino S., Kitamura A., Tomine Y., Tanaka I., Nishi M., Nonaka K., Nofuji Y., Narita M., Taniguchi Y., Yokoyama Y. (2019). A Community-Wide Intervention Trial for Preventing and Reducing Frailty Among Older Adults Living in Metropolitan Areas: Design and Baseline Survey for a Study Integrating Participatory Action Research With a Cluster Trial. J. Epidemiol..

[26] Taniguchi Y., Seino S., Nishi M., Tomine Y., Tanaka I., Yokoyama Y., Amano H., Kitamura A., Shinkai S. (2018). Physical, social, and psychological characteristics of community-dwelling elderly Japanese dog and cat owners. PLoS ONE.

[27] Nakanishi M., Nakashima T. (2014). Features of the Japanese national dementia strategy in comparison with international dementia policies: How should a national dementia policy interact with the public health- and social-care systems?. Alzheimer’s Dement. J. Alzheimer’s Assoc..

[28] Tomata Y., Sugiyama K., Kaiho Y., Honkura K., Watanabe T., Zhang S., Sugawara Y., Tsuji I. (2016). Green Tea Consumption and the Risk of Incident Dementia in Elderly Japanese: The Ohsaki Cohort 2006 Study. Am. J. Geriatr. Psychiatry Off. J. Am. Assoc. Geriatr. Psychiatry.

[29] Nakazawa A., Nakamura K., Kitamura K., Yoshizawa Y. (2012). Association between activities of daily living and mortality among institutionalized elderly adults in Japan. J. Epidemiol..

[30] Taniguchi Y., Kitamura A., Murayama H., Amano H., Shinozaki T., Yokota I., Seino S., Nofuji Y., Nishi M., Yokoyama Y. (2017). Mini-Mental State Examination score trajectories and incident disabling dementia among community-dwelling older Japanese adults. Geriatr. Gerontol. Int..

[31] Taniguchi Y., Kitamura A., Seino S., Murayama H., Amano H., Nofuji Y., Nishi M., Yokoyama Y., Shinozaki T., Yokota I. (2017). Gait Performance Trajectories and Incident Disabling Dementia Among Community-Dwelling Older Japanese. J. Am. Med. Dir. Assoc..

[32] Taniguchi Y., Kitamura A., Kaito S., Yokoyama Y., Yokota I., Shinozaki T., Seino S., Murayama H., Matsuyama Y., Ikeuchi T. (2019). Albumin and Hemoglobin Trajectories and Incident Disabling Dementia in Community-Dwelling Older Japanese. Dement. Geriatr. Cogn. Disord..

[33] Noda H., Yamagishi K., Ikeda A., Asada T., Iso H. (2018). Identification of dementia using standard clinical assessments by primary care physicians in Japan. Geriatr. Gerontol. Int..

[34] Ikeda A., Yamagishi K., Tanigawa T., Cui R., Yao M., Noda H., Umesawa M., Chei C., Yokota K., Shiina Y. (2008). Cigarette smoking and risk of disabling dementia in a Japanese rural community: A nested case-control study. Cerebrovasc. Dis..

[35] Yamamoto T., Kondo K., Hirai H., Nakade M., Aida J., Hirata Y. (2012). Association between self-reported dental health status and onset of dementia: A 4-year prospective cohort study of older Japanese adults from the Aichi Gerontological Evaluation Study (AGES) Project. Psychosom. Med..

[36] Ministry of Health, Labour and Welfare The Current Situation and the Future Direction of the Long-Term Care Insurance System in Japan. http://www.mhlw.go.jp/english/policy/care-welfare/care-welfare-elderly/dl/ri_130311-01.pdf.

[37] Taniguchi Y., Kitamura A., Shinozaki T., Seino S., Yokoyama Y., Narita M., Amano H., Matsuyama Y., Fujiwara Y., Shinkai S. (2018). Trajectories of arterial stiffness and all-cause mortality among community-dwelling older Japanese. Geriatr. Gerontol. Int..

[38] Taniguchi Y., Kitamura A., Nofuji Y., Ishizaki T., Seino S., Yokoyama Y., Shinozaki T., Murayama H., Mitsutake S., Amano H. (2019). Association of Trajectories of Higher-Level Functional Capacity with Mortality and Medical and Long-Term Care Costs Among Community-Dwelling Older Japanese. J. Gerontol. A Biol. Sci. Med. Sci..

[39] Yoshizaki T., Yokoyama Y., Oue A., Kawaguchi H. (2019). Association of Dietary Variety with Nutrient and Food Group Intake and Frailty among Community-dwelling Japanese Older Adults. Jpn. J. Nutr. Diet..

[40] Guralnik J.M., LaCroix A.Z., Abbott R.D., Berkman L.F., Satterfield S., Evans D.A., Wallace R.B. (1993). Maintaining mobility in late life. I. Demographic characteristics and chronic conditions. Am. J. Epidemiol..

[41] Kinugasa T., Nagasaki H. (1998). Reliability and validity of the Motor Fitness Scale for older adults in the community. Aging.

[42] Shinkai S., Yoshida H., Taniguchi Y., Murayama H., Nishi M., Amano H., Nofuji Y., Seino S., Fujiwara Y. (2016). Public health approach to preventing frailty in the community and its effect on healthy aging in Japan. Geriatr. Gerontol. Int..

[43] Saito M., Kondo K., Ojima T., Hirai H. (2015). Criteria for social isolation based on associations with health indicators among older people. A 10-year follow-up of the Aichi Gerontological Evaluation Study. Nihon Koshu Eisei Zasshi Jpn. J. Public Health.

[44] Hoyl M.T., Alessi C.A., Harker J.O., Josephson K.R., Pietruszka F.M., Koelfgen M., Mervis J.R., Fitten L.J., Rubenstein L.Z. (1999). Development and testing of a five-item version of the Geriatric Depression Scale. J. Am. Geriatr. Soc..

[45] Awata S., Bech P., Yoshida S., Hirai M., Suzuki S., Yamashita M., Ohara A., Hinokio Y., Matsuoka H., Oka Y. (2007). Reliability and validity of the Japanese version of the World Health Organization-Five Well-Being Index in the context of detecting depression in diabetic patients. Psychiatry Clin. Neurosci..

[46] Angrist J.D., Krueger A.B. (2001). Instrumental Variables and the Search for Identification: From Supply and Demand to Natural Experiments. J. Econ. Perspect..

[47] Koohsari M., Nakaya T., McCormack G., Shibata A., Ishii K., Yasunaga A., Liao Y., Oka K. (2020). Dog-walking in dense compact areas: The role of neighbourhood built environment. Health Place.

[48] Sado M., Ninomiya A., Shikimoto R., Ikeda B., Baba T., Yoshimura K., Mimura M. (2018). The estimated cost of dementia in Japan, the most aged society in the world. PLoS ONE.

[49] Hanaoka S., Matsumoto K., Kitazawa T., Fujita S., Seto K., Hasegawa T. (2019). Comprehensive cost of illness of dementia in Japan: A time trend analysis based on Japanese official statistics. Int. J. Qual. Health Care.

[50] Ikeda S., Mimura M., Ikeda M., Wada-Isoe K., Azuma M., Inoue S., Tomita K. (2021). Economic Burden of Alzheimer’s Disease Dementia in Japan. J. Alzheimer’s Dis. JAD.

[51] Shiroiwa T., Noto S., Fukuda T. (2021). Japanese Population Norms of EQ-5D-5L and Health Utilities Index Mark 3: Disutility Catalog by Disease and Symptom in Community Settings. Value Health.

[52] Shearer J., Green C., Ritchie C.W., Zajicek J.P. (2012). Health state values for use in the economic evaluation of treatments for Alzheimer’s disease. Drugs Aging.

[53] Japan Pet Food Association https://petfood.or.jp/pdf/data/2023/3.pdf.

[54] Shiroiwa T., Igarashi A., Fukuda T., Ikeda S. (2013). WTP for a QALY and health states: More money for severer health states?. Cost Eff. Resour. Alloc..

[55] Shiroiwa T., Fukuda T., Ikeda S., Takura T., Moriwaki K. (2017). Development of an Official Guideline for the Economic Evaluation of Drugs/Medical Devices in Japan. Value Health.

[56] Taniguchi Y., Kobayashi M. (2025). Association of new dog ownership with physical activity and social contact: The retrospective study. Sci. Rep..

[57] National Institute of Health and Nutrition https://www.nibn.go.jp/eiken/english/research/pdf/epar2006.pdf.

[58] Soares J., Epping J.N., Owens C.J., Brown D.R., Lankford T.J., Simoes E.J., Caspersen C.J. (2015). Odds of Getting Adequate Physical Activity by Dog Walking. J. Phys. Act. Health.

[59] Carr D., Friedmann E., Gee N.R., Gilchrist C., Sachs-Ericsson N., Koodaly L. (2021). Dog Walking and the Social Impact of the COVID-19 Pandemic on Loneliness in Older Adults. Animals.

[60] World Health Organization (2019). Risk Reduction of Cognitive Decline and Dementia: WHO Guidelines.

[61] Handlin L., Hydbring Sandberg E., Nilsson A., Ejdeback M., Jansson A., Uvnäs-Moberg K. (2011). Short-Term Interaction between Dogs and Their Owners: Effects on Oxytocin, Cortisol, Insulin and Heart Rate—An Exploratory Study. Anthrozoös.

[62] Nagasawa M., Mitsui S., En S., Ohtani N., Ohta M., Sakuma Y., Onaka T., Mogi K., Kikusui T. (2015). Oxytocin-gaze positive loop and the coevolution of human-dog bonds. Science.

[63] Gupta S. (2017). Microbiome: Puppy power. Nature.

[64] Seino S., Hata T., Mori H., Shinkai S., Fujiwara Y., Kobayashi E. (2025). Impact of the COVID-19 Pandemic on New Long-term Care Insurance Applications and All-cause Mortality in Older Adults in a Japanese Metropolitan Cohort: An Interrupted Time-series Analysis. J. Epidemiol..

