# Supplementary material for: Investigating the Potential Protective Effect of Dog Ownership on Incident Disabling Dementia and Its Cost-Effectiveness
Source: Int J Environ Res Public Health. 2026 Jul 22;23(7):938. doi: 10.3390/ijerph23070938 (PMC13410369; doi:10.3390/ijerph23070938)
Supplement: Supplementary file 1 [file ijerph-23-00938-s001.zip › ijerph-4342313-supplementary.pdf]

Supplementary Materials

Title: Investigating the potential association of dog ownership with disabling dementia, mortality, and cost-effectiveness in older Japanese adults.

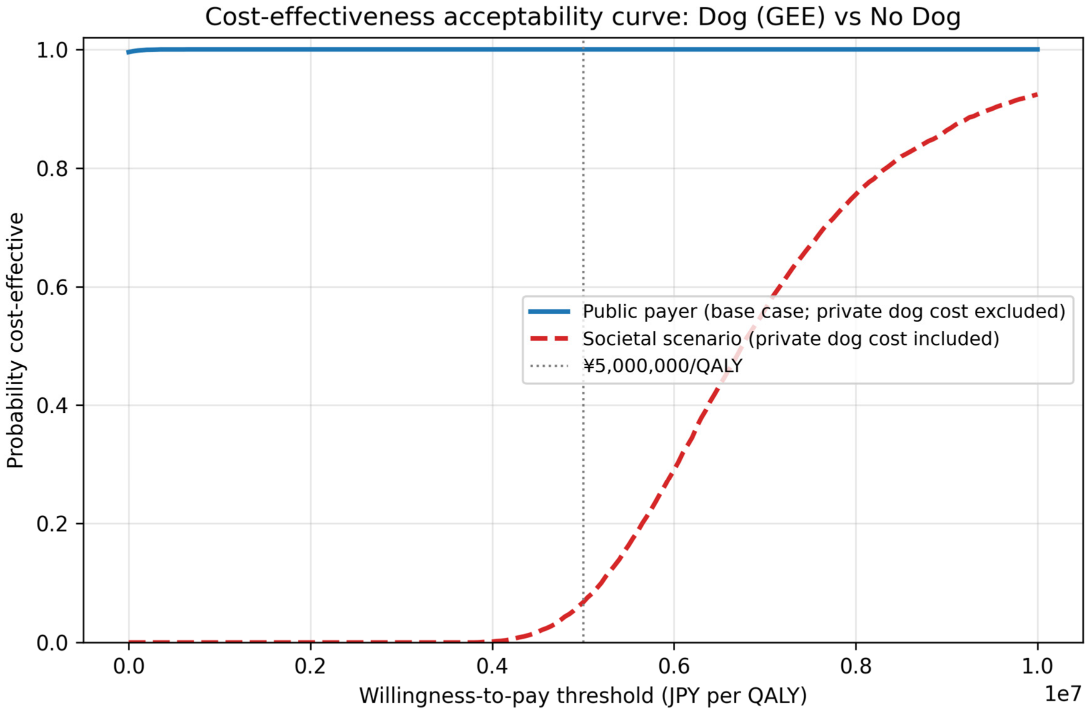

Figure S1. Cost-effectiveness acceptability curve for the base-case comparison of dog ownership (Dog (GEE)) versus no dog, derived from 10,000 first-order Monte Carlo simulations of the Markov model, under two perspectives. Solid line: public payer perspective (base case; private dog-ownership costs excluded), under which dog ownership was cost-saving and cost-effective with a probability of approximately 100% at any willingness-to-pay threshold (about 99% even at a threshold of zero, reflecting net cost saving). Dashed line: societal scenario (private dog-ownership costs included), under which the probability of being cost-effective at ¥5,000,000 per QALY was approximately 7%. The dotted vertical line marks the ¥5,000,000 per QALY threshold.

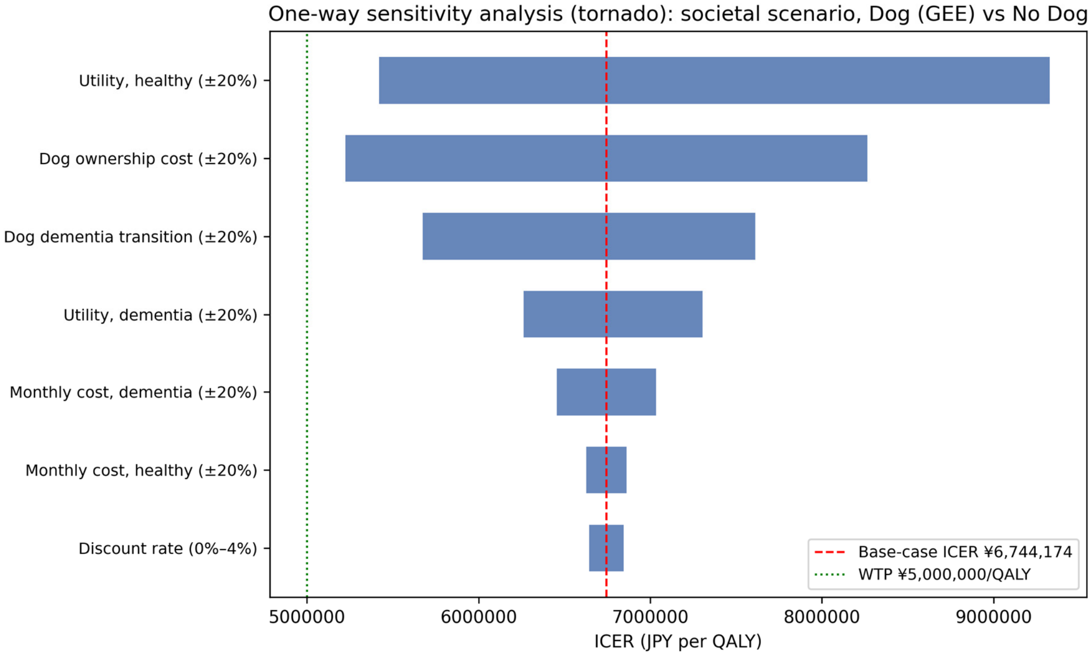

Figure S2. One-way sensitivity analysis (tornado diagram) of the incremental cost-effectiveness ratio (ICER) for the Dog (GEE) scenario versus no dog in the societal scenario that includes private dog-ownership costs (the analysis in which cost-effectiveness is threshold-dependent; in the base-case public payer analysis dog ownership was dominant). Each bar shows the range of the ICER when a single parameter is varied over the indicated range (health-state costs and utilities ±20%, dog-ownership cost ±20%, discount rate 0–4%, and the dog-related dementia transition probability ±20%). The red dashed line is the base-case ICER and the green dotted line is the ¥5,000,000 per QALY threshold.
